# Decoding p53 tumor suppression: a crosstalk between genomic stability and epigenetic control?

**DOI:** 10.1038/s41418-024-01259-9

**Published:** 2024-02-20

**Authors:** Ana Janic, Etna Abad, Ivano Amelio

**Affiliations:** 1 Department of Medicine and Life Sciences, Universitat Pompeu Fabra, Barcelona, Spain; 2 Chair for Systems Toxicology, University of Konstanz, Konstanz, Germany

## Abstract

Genomic instability, a hallmark of cancer, is a direct consequence of the inactivation of the tumor suppressor protein p53. Genetically modified mouse models and human tumor samples have revealed that p53 loss results in extensive chromosomal abnormalities, from copy number alterations to structural rearrangements. In this perspective article we explore the multifaceted relationship between p53, genomic stability, and epigenetic control, highlighting its significance in cancer biology. p53 emerges as a critical regulator of DNA repair mechanisms, influencing key components of repair pathways and directly participating in DNA repair processes. p53 role in genomic integrity however extends beyond its canonical functions. p53 influences also epigenetic landscape, where it modulates DNA methylation and histone modifications. This epigenetic control impacts the expression of genes involved in tumor suppression and oncogenesis. Notably, p53 ability to ensure cellular response to DNA demethylation contributes to the maintenance of genomic stability by preventing unscheduled transcription of repetitive non-coding genomic regions. This latter indicates a causative relationship between the control of epigenetic stability and the maintenance of genomic integrity in p53-mediated tumor suppression. Understanding these mechanisms offers promising avenues for innovative therapeutic strategies targeting epigenetic dysregulation in cancer and emphasizes the need for further research to unravel the complexities of this relationship. Ultimately, these insights hold the potential to transform cancer treatment and prevention strategies.

## Facts

- p53 safeguards genomic stability by participating in DNA repair and epigenetic regulation.
- In cancer, p53 inactivation elicits extensive genomic instability with an evident preferred order of genomic aberrations.
- Loss of p53 creates susceptibility to epigenetic perturbations, leading to genomic instability.
- Gain-of-function p53 mutants can manipulate epigenetic regulators and modify the chromatin landscape.

## Open questions

- What are the individual contributions of p53-mediated epigenetic regulation and DNA repair mechanisms to its tumor-suppressive functions?
- How does p53-mediated metabolic regulation influence the complex interplay between epigenetics and genomic stability?
- Can we decipher the specific p53-dependent DNA repair pathways to formulate targeted therapies and capitalize on vulnerabilities?
- Is it possible to transform our understanding of the interplay between p53, genomic integrity, and epigenetics into diagnostic or prognostic tools for personalized cancer management?

## Introduction

Inactivated in approximately half of all human cancers, the tumor suppressor protein TP53 (hereafter p53) plays a crucial role in maintaining genomic stability [1, 2]. The *p53* gene comprises 11 exons, spanning ~20 kilobases and encodes a protein consisting of 393 amino acids [3]. This protein serves as a transcription factor, activating or repressing the expression of a myriad of target genes involved in diverse cellular processes [4]. The intricate orchestration of p53 tumor-suppressive functions relies on a finely tuned balance of its synthesis, posttranslational modification, stabilization, and degradation [5]. In normal, unstressed conditions, the levels of p53 protein are generally attenuated by the actions of MDM2, the dedicated ubiquitin ligase targeting p53. MDM2 binds to p53 in its transactivation domain regions, immediately impacting on its transcriptional activity and promoting it for proteasomal degradation [6, 7]. However, in response to cellular stress, such as DNA damage or oncogenic signaling, p53 is activated through a complex series of post-translational modifications, including phosphorylation, acetylation, and sumoylation, which disrupt its interaction with MDM2. Consequently, p53 levels increases, and through binding to specific DNA consensus sequences p53 executes its critical tumor-suppressive functions [8].

While it is well-accepted that the cooperation of a range of cell-autonomous and nonautonomous functions underlies p53 tumor suppression capacity, the ability to prevent genomic instability and preserve quality of the genetic information appears a primary role for p53 [9]. Induction of p53-dependent cell cycle arrest, apoptosis, or senescence, in response to DNA damage was postulated to be the main process for suppressing mutagenesis and tumor development [10, 11]. The importance of these mechanisms, however varies depending on the cell type and oncogenic lesions and other non-canonical processes appear essentially involved in its genomic stability regulations [12, 13].

Genomic instability in cancer emerges from multiple, substantially different, processes [14]. These include defects in DNA repair, epigenetic alterations, and aberrant mitosis. Recent evidences have implicated p53 in several of these processes, accounting for potential alternative mechanisms underlying the genomic instability observed in p53 mutated cancers [15]. Errors in mitosis can result in chromosome missegregation, chromosomal rearrangements, and aneuploidy, which are main cause of genomic evolution of cancers [16, 17]. In response to DNA damage, p53 can induce the arrest of cellular proliferation, allowing cells time to repair damaged DNA and preventing further proliferation of damaged cells, thus avoiding the propagation of mutations. Epigenetic alterations involve modifications to the structure and function of DNA without involving effective mutagenesis. These alterations influence gene expression as well as regulation of heterochromatin regions impacting on genomic instability [18]. DNA repair mechanisms playdirect crucial regulations in preventing mutagenesis. Defects in these repair mechanisms produce upon DNA damage accumulation of genetic alterations promoting mutagenesis.

Collectively, defects in DNA repair, epigenetic alterations, and aberrant mitosis contribute from alternative directions to genomic instability, promoting initiation and progression of cancer. In this perspective we will summarize the recent evidence implicating p53 in these processes, pointing out the importance of its role in the coordination of quality controls mechanisms to ensure genomic integrity. Specifically, within this, we explore the postulation that a close interplay between p53 control of genomic integrity and epigenetic regulations exists. A deeper understanding of these mechanisms, their interplay and their interactions with p53 inactivation might support development of alternative strategies to target p53 deficient tumors, converting genomic instability into a cancer vulnerability. The delineation of the underlying causes of p53 associated genomic instability can also support preventive measures for cancer.

## p53 loss and genomic instability: a close relationship

In human cancers, when p53 is defective or absent, cells continue to divide uncontrolled, giving rise to the cells with extensive genomic abnormalities [19]. These aberrations might include single nucleotide variants, polyploidies, large-scale structural re-arrangements that in some cases, could resemble chromotrypsis. Compelling in vivo evidence supporting the idea that genomic instability is one of the primary drivers of the tumorigenesis in the absence of p53 comes from various studies on p53 deficient mice [15]. For example, tumors derived from p53^+/−^ heterozygous mice, in which the wild-type allele has been deleted, displayed a higher frequency of chromosomal copy number alterations compared to tumors that retained one copy of the p53 allele [20]. Using whole genome sequencing, it has been observed a number of large-scale copy number changes in Myc lymphomas driven by the knockdown of p53 [21]. Mice deficient for p53 are developmentally normal but susceptible to spontaneous tumors [22]. Similarly in the T-cell lymphomas from p53 knockout mice have shown very high rate of gene copy number variations, including chromotrypsis-like events and alteration of the genes that have been identified as known drivers of the T-cell lymphomas in humans, including PTEN, CDK6, or RB [23]. Likewise, in humans, upon analyzing TCGA cancer types, a substantial number of tumors carrying p53 mutations exhibited significantly higher levels of copy number changes in the p53 mutant cancer group compared to their wild-type p53 counterparts. This extensive chromosomal instability was strongly correlated with increased amplification of well-known oncogenes and/or deletion of tumor suppressor genes (such as RB1 and PTEN) [24]. Notably, increase in structural variants, such as deletions and duplications, in metastatic tumors have been strongly linked to p53 mutations across different types of cancer [25, 26]. Altogether, this supports the notion that p53-deficient cancers have chromosomal instability, rather than single nucleotide (or small DNA segment) instability.

**Fig. 1:**
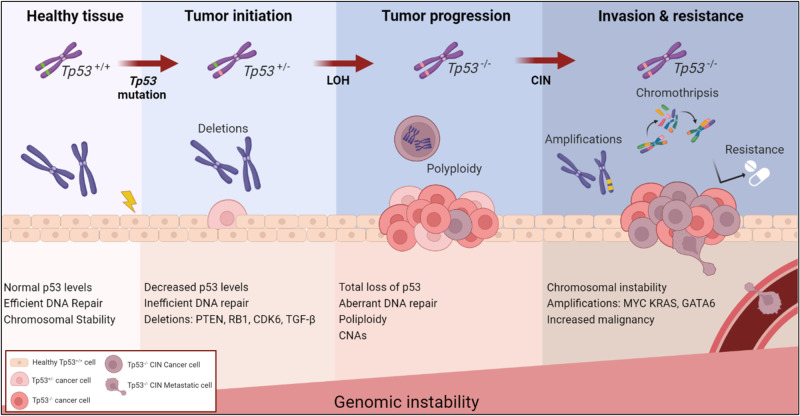
Loss of functional p53 is a critical factor in promoting genomic instability and contributing to cancer development. The sequence and timing of the events in the development of genomic instability have a defined order. Schematic representation of order in genetic events proposed to accompany the transition from normal tissue to malignancy. Created with BioRender.com (Agreement number: IR26AKIJ23).

A recent study utilizing genetically modified mouse models and human tumor samples of pancreatic cancer has revealed that the absence of *Trp53* absence does not simply lead to “genetic chaos” but can instead drive accumulation of non-random genetic events from pre-malignant to full-blown tumors [27]. In this study in the context of pancreatic cancer authors have shown that the earliest event after p53 inactivation are deletions in certain pathways that are essential for malignant initiation (e.g., TGF-b signaling, chromatin remodeling, axon guidance). This is followed by polyploidy and as a tumor progresses there is gradual accumulation of gains and amplifications, such as Myc, Kras, or GATA6 (Fig. 1). This research highlights the importance of continuous and ordered chromosomal instability throughout tumor progression. Similarly, it has been shown in gastric cancers that p53 loss induces aneuploidy, marked by the ordered copy number and structural variations changes [28]. Gaining a deeper understanding of the progression of genetic lesions and how they fictionally contribute to malignancy, will have significant implications for developing effective treatments and strategies for cancer prevention.

## p53-mediated DNA repair

**Fig. 2:**
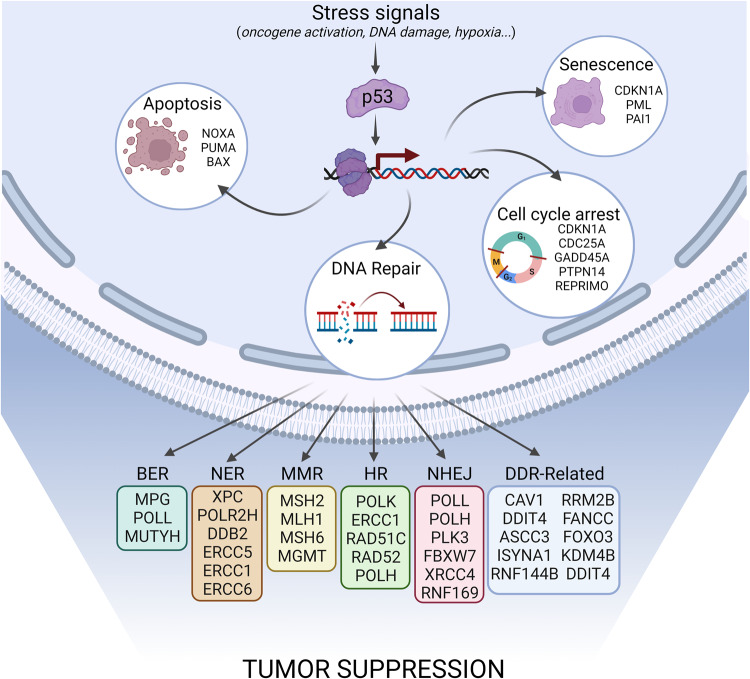
Activation of p53 in response to stress signals initiates its transcriptional activity, leading to the activation of cellular protective pathways. p53 binds to the DNA in a tetrameric configuration and promotes the transcription of a wide array of genes. Pictured are key p53 pathways and transcriptional targets regulated by p53 with a specific emphasis on p53-dependent DNA repair genes. BER (base excision repair), NER (nucleotide excision repair), MMR (mismatch repair), HR (homologous recombination), NHEJ (non-homologous end-joining), DDR (DNA damage repair) Created with BioRender.com (Agreement number: YM26AKJ2TU).

Cancer is caused by the gradual accumulation of genetic mutations through different mechanisms, such as epigenetic modifications and DNA repair. In order to secure normal performance and preserve genome integrity, cells have acquired a set of the DNA damage repair mechanisms, as nucleotide excision repair, base excision repair, mismatch repair, homologous recombination, and non-homologous end-joining. It has been known already for more than 30 years that p53 acting as the “guardian of the genome” has a critical role in maintaining genome integrity [2]. Upon DNA damage or oncogene activation, p53 transcriptionally controls essential components of the various DNA damage repair machineries, including Msh2, XRCC4, Rad51, Polk, Ercc5, Mgmt, Ku86, Xpc, Ddb2, or Gadd45α [29, 30]. Moreover, it is known that p53 directly controls different forms of DNA repair [9] (Fig. 2).

In the last decade, numerous examples have emerged about the importance of p53 in modulating DNA repair mechanisms to promote tumor suppression. Identifying the molecular mechanisms underlying p53 function in tumor suppression is crucial for understanding cancer development, and genetically modified mouse models have provided a useful tool for advancing this research question. Targets of p53 that promote apoptosis, cell cycle arrest, and cell senescence have been considered as essential effectors of p53-mediated tumor suppression (Fig. 2). However, mice deficient for any single one of the p53 target genes involved in apoptosis, cell cycle arrest or cell senescence (e.g., Puma, Noxa, p21, or Gadd45a) fail to recapitulate the dramatic and fully penetrant spontaneous tumor predisposition of p53-deficient mice. Further, and remarkably, no spontaneous tumors arise in mice lacking PUMA, Noxa, and p21, the critical mediators of p53-induced apoptosis and G1/S cell cycle arrest/senescence [31–33]. Likewise, p53 mutants that are defective in the induction of both apoptosis and cell-cycle arrest, or in all three responses (apoptosis, cell-cycle arrest, and senescence) retain tumor suppressor activity [13, 34]. What, then are the most important p53 activities need for tumor suppression? [9] More recently, functional genomic screens in vivo in mouse hematopoietic cells with sensitized background identified p53 target genes whose knock-down can promote blood cancer development. In those pooled screenings more then 160 known p53 downstream target genes have been tested, and genes involved in DNA repair and cell proliferation, as well as some with poorly defined functions have been identified as most potent hits. Interestingly, disruption of several direct p53-activated target genes implicated in DNA damage repair, including Mlh1, Msh2, Polk, Rnf144b, Ddit4, and FancC accelerated Myc oncogene driven lymphomagenesis, mirroring the effect of Trp53 loss [21]. These studies indicate that effective coordination of DNA repair plays a critical role in p53-mediated tumor suppression, particularly in the context of blood cancers. Testing the importance of DNA repair factors downstream of p53 in tumor suppression in broader range of tissues or cancer settings will allow further assessment of the generality of these findings.

## p53-mediated regulations of DNA methylation: a key for genomic instability?

Functioning as a transcriptional factor p53 has a direct impact on site-specific epigenetic regulations. p53 can physically interact with DNA and histone methyltransferases. By directly binding to DNA methyltransferases or indirectly affecting their expression, p53 can promote DNA demethylation or inhibit de novo DNA methylation at specific genomic loci [35, 36]. However, the diversity of chromatin features at p53 transcription factor binding sites does not depend on DNA methylation. Instead, a cofactor called Trim24 interacts with p53 and unmethylated histone 3 lysine 4 (H3K4), guiding p53 to closed chromatin sites. This connection between H3K4 methylation and p53 function reveals how chromatin specificity is achieved through cofactors that locally influence transcription factor activity [37].

This dynamic control of epigenetic methylation by p53 contributes to the regulation of key genes involved in cell tumor suppressor pathways and oncogenic processes [38]. In addition to the site-specific epigenetic regulations produced at the p53 responsive genomic site, p53 orchestrates alterations in the metabolic landscape, instigating modifications in chromatin structure and gene expression. Upon restoring p53 function in cancer cells derived from KRAS-mutant mouse models of PDAC, there is an observed augmentation in the accumulation of α-ketoglutarate, a metabolite crucial as a critical substrate for demethylating chromatin enzymes. The transcriptional programs induced by p53 exhibit characteristics indicative of premalignant differentiation, a phenomenon partially mimicked by the introduction of cell-permeable α-ketoglutarate. The escalation of the α-ketoglutarate-dependent product of cytosine demethylation, 5-hydroxymethylcytosine (5hmC), accompanies p53-triggered tumor cell differentiation. In contrast, the transition from premalignant to de-differentiated malignant lesions, associated with Trp53 mutations, is marked by diminished 5hmC. Inhibition of enzymes capable of producing α-ketoglutarate accumulation specifically results in increased 5hmC, tumor cell differentiation, and reduced tumor cell fitness [39]. These findings implicate α-ketoglutarate in p53-mediated tumor suppression, suggesting that the buildup of α-ketoglutarate in p53-deficient tumors may drive tumor cell differentiation while counteracting cancer progression.

**Fig. 3:**
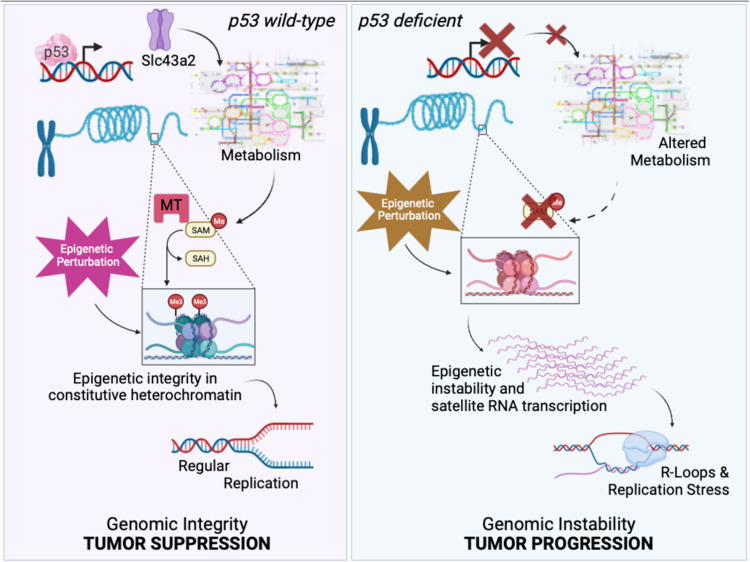
p53 is a critical factor in controlling the epigenetic integrity of constitutive heterochromatin, preventing genomic instability. Cells lacking functional p53 display altered metabolic status, including a reduction in S-Adenosyl-Methionine (SAM) levels. The reduced availability of SAM impairs the cell’s ability to respond to epigenetic perturbations, as SAM is an essential precursor for the reactions catalyzed by multiple methyl-transferases (MT) that control DNA and histone methylation. The consequential effect of this deregulation is epigenetic instability of constitutive heterochromatin and transcription of repetitive sequences within these genomic regions. Uncontrolled transcription of repetitive sequences disrupts replication progression, leading to genomic instability. Modified from Panatta et al. [40].

A close relationship between p53 function and the regulation of epigenetic-metabolism crosstalk emerges also in the analysis of the response of p53-deficient cells to exogenous perturbation of DNA methylation. A recent study reported how p53 loss exposes the cells to inability to effectively respond to DNA hypomethylation. p53 inactivation impacts the level of S-adenosylmethionine, the major methyl-donor required for epigenetic methylation, and its consequences on epigenetic regulation and genomic stability [40]. Mimicking the DNA demethylation produced by various stressors with a hypomethylating agent, p53-deficient cells appeared to have insufficient SAM levels to preserve stability of repressive H3K9me3 histone modification. This instability triggers the transcription of repetitive satellite DNA due to the relaxation of constitutive heterochromatin (Fig. 3). The aberrant transcriptional control of noncoding repetitive regions results in the accumulation of an unscheduled R-loop, leading to replication stress, transcription-replication conflicts, and chromosomal abnormalities. S-adenosylmethionine is produced within the one-carbon metabolism, a metabolic process that appears to be under the control of p53. p53 transcriptionally regulates a set of genes involved in one-carbon metabolism, including the methionine transporter Slc43a2. Depletion of Slc43a2 recapitulates the effects of p53 loss, while its reintroduction in a p53-depleted background reverses these effects. Slc43a2 manipulation displays altered transcriptional control of noncoding repetitive regions with alterations of unscheduled R-loop accumulations, and chromosomal abnormalities. Deregulation of Slc43a2 has been associated with altered methionine metabolism and histone methylation in cancer. Slc43a2 expression also appeared correlated with p53 status, and the prognosis of pancreatic ductal adenocarcinoma (PDAC) patients, providing clinical relevance to these findings and opening to an intriguing correlation between genomic instability and regulation of one-carbon metabolism [40]. Consistently, previous studies reported that in cooperation with DNA methylation, p53 represses expression of repetitive and transposon sequences, suggesting a link between repetitive sequences and loss of genome integrity in p53 inactivated tumorus [41]. Similarly, derepression of transposon elements, such as LINE1, was observed in Drosophila lacking the p53-family ortholog Dmp53. Dmp53 occupies 5′UTR of L1s and establishing deposition of repressive histone marks H3K9me3 and transcriptional suppression of these elements [42]. The data indicate the existence of an evolutionary conserved mechanism of heterochromatin preservation by p53, that has further evolved in vertebrates with the co-operation of histone and DNA methylation. Consistently with this, p53-dependent R-loops were reported to accumulate in High-risk human papillomaviruses-driven cancers, participating in the etiology and pathogenesis of the disease [43]. The effective contribution of the p53-mediated control of epigenetic stability in the tumor suppression remains however to be determined. Intriguingly, however, accumulation of repetitive RNAs as well as genomic instability associated cytoplasmic DNA can trigger an indication of type I interferon response. p53 function has been indeed associated with both positive and negative effects on the cGAS/STING-mediated induction of type I interferon response [41, 44].

Genomic integrity and epigenetic control are directly implicated in the regulatory processes that govern nuclear architecture [45]. Loss of p53 increases lamin B1 expression, a critical component of the nuclear envelope. This regulation impacts chromatin accessibility and gene expression in pancreatic cancer, revealing a potential connection with p53-nuclear architecture. Remarkably, p53-depedent changes in chromatin accessibility not only correlate with changes in gene expression but also with high frequencies of genomic rearrangements of these genes in pancreatic cancer patients [46] (Fig. 3).

The intriguing relationship between genomic integrity and epigenetics in p53 tumor suppression is ripe for further exploration, yielding a plethora of unanswered questions. One pivotal inquiry revolves around the extent to which p53-mediated metabolic regulations influence epigenetic processes and genomic stability. This area represents an emerging frontier in p53 research.

## p53-mediated epitranscriptomic regulations

The intricate landscape of p53-mediated cellular responses extends beyond its roles in epigenetic regulations, encompassing emerging realms such as the regulation of epitranscriptomics. Epitranscriptomics refers to sum of the chemical modifications that occur on RNA molecules, such as methylation, pseudouridylation, etc, that can dynamically regulate various aspects of RNA function, including stability, splicing and transport [47]. Recent advances reveal that p53 plays a role in shaping the epitranscriptomic landscape through dynamic interactions with RNA-modifying enzymes and regulatory pathways. Using tandem affinity purification/mass spectrometry to identify p53-interacting proteins, Attardi and colleagues revealed that p53 physically interact with the epitranscriptomic writer, METTL3, thus amplifying the p53-mediated stress responses. METTL3 is a constituent of the m^6^A RNA-methyltransferase complex, that in response to DNA damage and oncogenic signals promotes stabilization of p53 protein and expression of target genes. Remarkably, the tumor suppressive capacity of METTL3, assessed in in vivo mouse transplanted cancer models, is observed only in p53 proficient condition [48]. The interplay between p53 and key epitranscriptomic effectors, including writers, erasers, and readers, is still to be explored and elucidated. Epitranscriptomic regulations have significance for regulation of genomic integrity, however whether there is an implication for p53-mediated control of genomic stability is still not clear [49].

## p53 gain-of-function mutant and epigenetic regulations

The mutations in p53 generally inactivate its tumor suppression, leading to a loss of function effect. The largest proportion of these mutations leads, however to expression of p53 mutant proteins that have been postulated to exert direct pro-tumorigenic roles via dominant negative effects as well as gain-of-function (GOF) effects, including via regulation of cancer cells epigenetic landscape [50–52]. Remarkably, the largest majority of p53 GOF mutants display alterations in their DNA binding domain, thus, despite they have been described to still display capability to interact with the chromatin and influence transcription, they do not conserve the DNA binding profile of the p53 wt protein.

The p53 status has a direct impact on epigenetic pattern of cancer cells. Cancer that preserves unmutated p53, for example, can activate alternative epigenetic mechanisms to influence expression of p53 downstream targets. In p53 wild-type glioblastoma (GBM), p53 tumor-suppressive function is hindered by the chromatin regulator BRD8. BRD8 maintains a repressive chromatin state at p53 target sites, preventing p53 activation and sustaining cell proliferation. Targeting BRD8 bromodomain displaces H2AZ, making chromatin accessible for p53 activation, leading to cell cycle arrest and tumor suppression, presenting a potential therapeutic avenue for p53 wt GBM [53].

**Fig. 4:**
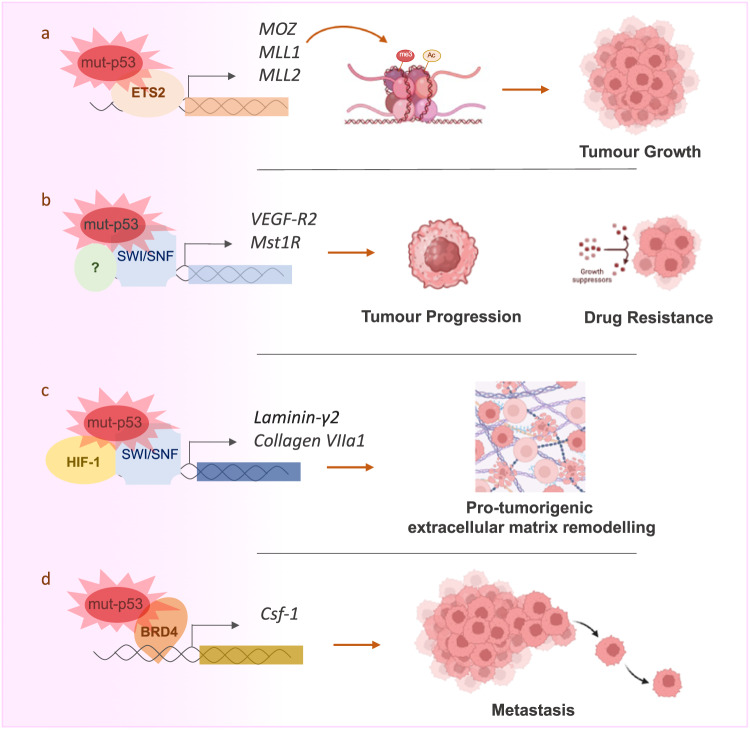
Mutant p53 alters the epigenetic landscape of cancer cells via multiple mechanisms. **a** The interaction between mutant p53 and ETS2 has been demonstrated to enhance the expression of epigenetic regulators, namely, MOZ, MLL1, and MLL2. The upregulation of these regulators is implicated in promoting tumor growth. **b**, **c** The interaction of mutant p53 with the SWI/SNF chromatin remodeling complex, involving HIF-1 or alternative unidentified transcriptional factors, induces the expression of genes that contribute to therapy resistance, microenvironment remodeling, and tumor progression. **d** Mutant p53 recruitment by BRD4 to the Csf-1 promoter, highly enriched in H3 Lysine 27 Acetylation, results in the induction of its expression, thereby promoting metastasis.

While wildtype p53 appears to influence enhancer and promoter landscape promoting regulation of its tumor suppressive programs [54], gain-of-function p53 mutant proteins hijack chromatin remodeling factors and epigenetic regulators promoting content-dependent pro-tumorigenic epigenetic regulations [55]. p53 GOF mutants have the ability to physically interact with the promoter of methyltransferases MLL1 (also known as KMT2A), MLL2 (also known as KMT2D), and acetyltransferase MOZ (also known as KAT6A or MYST3) and transcriptionally upregulate them. Thus, p53 GOF mutants modulate histone methylation and acetylation across the entire genome [56] (Fig. 4a). Moreover, p53 mutant R273H displays ability to influence hypoxic gene expression profile by facilitating recruitment of SWI/SNF chromatin remodeling complex on the hypoxic responsive elements [55, 57]. p53^R270H^-dependent transcriptomic analysis and chromatin accessibility (ATAC-seq) profiling uncovered a molecular connection between p53-driven drug tolerance and epigenetic regulations in pancreatic cancer [58]. p53^R270H^ (mouse version of p53^R273H^) selectively finely tunes chromatin accessibility, shaping a transcriptional program and affecting drug tolerance in cancer cells. In particular the tyrosine kinase receptor MST1r, whose chromatin accessibility and transcription are regulated in a p53^R270H^-mediated fashion, and significantly impacts drug response, mimicking the p53^R270H^-dependent phenotype. MST1r expression is associated with p53 mutant status and aggressive pancreatic cancer clinical features in patient analysis (Fig. 4b, c). Given that late-stage pancreatic cancer plasticity primarily arises from epigenetic mechanisms, this study proposed that mutant p53 contributes to the acquisition of a lethal phenotype by finely adjusting the chromatin landscape.

In esophageal squamous cell carcinoma the GOF mutant p53^R172H^ upregulates the colony-stimulating factor-1 (CSF-1) driving the lung metastatisation process. Cleavage Under Targets and Release Using Nuclease sequencing (CUT&RUN-seq) for H3K27ac, an active enhancer and promoter marker, in p53^R172H/−^ and p53^+/+^ cells revealed that more than 2000 genomic loci differentially enriched for this histone acetylation between the two conditions. H3K27ac was found enriched in the CSF-1 genomic locus, which also displayed enrichment in p53^R172H^ occupancy. The increase in H3K27ac fostered an interaction between bromodomain-containing domain 4 (BRD4) and p53-R172H to regulate *Csf-1* transcription (Fig. 4d). Thus, pharmacological inhibition of BRD4 was suggested as a vulnerability of p53^R172H^ expressing cancers [59]. Intriguingly, however a recent study showed that depletion by CRISPR/Cas9 of 12 distinct p53 mutants, known for their reported GOF effects displayed no discernible effects on the proliferation and response to chemotherapeutic agents in 15 human cancer cell lines and organoids, nor any effect in the capability of these cell lines to growth in vivo and colonize mouse organs [60]. The significance of the GOF effects mediated by the p53 mutant proteins remains, therefore, still highly debated [61].

## Conclusions

p53, regarded as the guardian of the genome, occupies a central position in the intricate interplay between cancer epigenetics and the maintenance of genomic integrity. Its multifaceted role encompasses both global and site-specific regulations within the cellular landscape. One critical consequence of p53 inactivation is the significant escalation of genomic instability, with an accumulation of a plethora of genetic abnormalities, spanning from single nucleotide variants to polyploidies and large-scale structural rearrangements, often resembling chromothripsis. However, p53 influence is not confined to DNA repair; it extends to epigenetics. It exerts control over DNA methylation and histone modifications, influencing the expression of genes vital for tumor suppression and oncogenic processes, as well as controlling critical part of the chromosome landscape, such as constitutive and facultative heterochromatin. This dynamic control of DNA methylation contributes to the regulation of key genes involved in preserving genomic stability. We argue that at the heart of p53 tumor-suppressive functions lie genomic integrity maintenance and epigenetic regulations, thus highlining the previously introduced thought-provoking concept of “p53 as the guardian of the epigenome” [38, 62]. The intricate connection between p53 roles in genomic integrity and epigenetics initiates a profound field of research and discussion. Understanding how p53 actions in DNA repair and epigenetic regulation intersect provides vital insights into the complex processes driving cancer development [63].

These insights hold the promise of innovative therapeutic strategies targeting epigenetic dysregulation in cancer and tumor specific DNA repair dependencies to preferentially kill cancer cells. At the same these concepts follow also the fascinating avenue of developing predictive tools based on epigenetic modifications to predict disease risk [64]. An additional emerging aspect is the role of p53 in epitranscriptomic regulations and their significance for the regulation of genomic integrity. Unraveling the intricacies of p53 involvement in epitranscriptomics would expand our understanding of p53 function and open new avenues for therapeutic interventions. Finally, the full extent of the significance of p53 GOF mutants remains unclear. A promising avenue for gaining insights into this matter could be achieved through a systematic assessment of how neomorphic p53 proteins react to physiological context-specific pressure. This approach not only responds to emerging evidence, indicating that understanding cancer progression requires delineating how extrinsic factors cooperate with oncogenic mutations [65–67], but also holds potential for reaching a consensus on the specific contentious issue of the p53 GOF effects. Once again, in these aspects, the interplay between genetics (specifically p53 mutational status) and existing microenvironmental signals emphasizes the potential importance of epigenetics in shaping our understanding of this complex issue. Hence, the crosstalk between p53, epigenetics, and genomic stability stands as an important axis to explore in our quest to unravel the bases of cancer and develop more effective treatments and preventive measures.
